# Androgen Receptor Localizes to Plasma Membrane by Binding to Caveolin-1 in Mouse Sertoli Cells

**DOI:** 10.1155/2017/3985916

**Published:** 2017-05-31

**Authors:** Qiong Deng, Yong Wu, Zeng Zhang, Yue Wang, Minghua Li, Hui Liang, Yaoting Gui

**Affiliations:** ^1^Department of Urology, People's Hospital of Longhua District, Shenzhen, China; ^2^Guangdong and Shenzhen Key Laboratory of Male Reproductive Medicine and Genetics, Institute of Urology, Peking University Shenzhen Hospital, Shenzhen PKU-HKUST Medical Center, Shenzhen, China; ^3^Department of Ultrasonic Imaging, Peking University Shenzhen Hospital, Shenzhen, China; ^4^Central Laboratory, Peking University Shenzhen Hospital, Shenzhen, China

## Abstract

The nonclassical androgen signaling pathway translates signals into alterations in cellular function within minutes, and this action is proposed to be mediated by an androgen receptor (AR) localized to the plasma membrane. This study was designed to determine the mechanism underlying the membrane association of androgen receptor in TM4 cells, a mouse Sertoli cell line. Western blot analysis indicated testosterone-induced AR translocation to the cell membrane. Data from coimmunoprecipitation indicated that AR is associated with caveolin-1, and testosterone enhanced this association. Knockdown of caveolin-1 by shRNA decreased the amount of AR localized to membrane fraction and prevented AR membrane trafficking after being exposed to testosterone at physiological concentration. The palmitoylation inhibitor 2-bromopalmitate decreased AR membrane localization in basal condition and completely blocked testosterone-induced AR translocation to membrane fraction. These data suggested that AR localized to membrane fraction by binding with caveolin-1 through palmitoylation of the cysteine residue. This study provided a new evidence for AR membrane localization and its application for clarifying the nonclassical signaling pathway of androgens.

## 1. Introduction

Male fertility depends on the successful perpetuation of spermatogenesis [1]. Spermatogenesis is supported by somatic Sertoli cells that surround and nurture the developing germ cells. Other hormones can facilitate this process; however, only the steroid hormone testosterone is essential to maintain spermatogenesis, and androgen receptor (AR) is the only specific receptor for androgens that has been identified.

In classical testosterone signaling, androgens exert their genomic effects via the AR [2]. When bound by androgens, cytoplasmic AR translocates to the nucleus and binds to androgen response elements within androgen-responsive genes [3]. This process is the classical signaling pathway of testosterone, which requires several hours to respond [3] since it relates to gene transcriptional activation or repression, as well as protein synthesis and secretion required to support spermatogenesis [4]. However, the genomic effect fails to adequately explain the regulation of spermatogenesis by androgens [5]. In addition to this effect, steroid hormone induces signaling events that originate at the surface of plasma membranes [4]. Studies demonstrate that functional AR is localized in the cell membrane [6]. Testosterone is associated with the membrane AR, and AR signaling translates signals into alterations in cellular function, thus maintaining spermatogenesis within minutes. The rapidly induced signaling effects of steroid hormones mediated via membrane-associated receptors are referred to as nonclassical effects [7]. This effect of testosterone involves activation of kinase signaling pathways and alteration in the expression of genes that have no known AREs or are not dependent on AR-promoter interactions [8, 9]. Our data showed that testosterone induced AR to the membrane in TM4 cells, a mouse Sertoli cell line. Nevertheless, the mechanism underlying the translocation of AR to the membrane remains unclear.

It was reported that palmitoylation of a cysteine in the conserved region of estrogen receptor (ER) increases the physical association of ER with caveolin-1 [10], which is the main component of the caveolae in the plasma membranes found in most cell types. This protein-protein interaction was required for membrane localization. Caveolin-1 null cancer cells that contain endogenous ER only show a nuclear pool of the receptor, and ER translocates to the plasma membrane upon introduction of caveolin-1 [11].

In the present study, we further identified the association of AR with caveolin-1 in the membrane fraction. Knockdown of caveolin-1 decreased AR localization in the plasma membrane and inhibited AR translocation to the membrane, induced by testosterone. Preincubation with the palmitoylation inhibitor prevented AR membrane trafficking. These data suggested that testosterone induced AR membrane association with caveolin-1 by palmitoylation.

## 2. Materials and Methods

### 2.1. Plasmid and Real-Time PCR Primers

Constructs for transient overexpression of shRNA targeted to caveolin-1 (GV-248) or control were obtained from Shanghai Genechem Co. Ltd. (Shanghai, China). The sequence of the shRNA is shown in Table 1. The insert sequence of the control shRNA was “TTCTCCGAACGTGTCACGT.”

Primers used for the mouse gene *Cav1* (132 bp) (Genebank: NM_007616): F 5′-ATG TCT GGG GGC AAA TAC GTG-3′, R 5′-CGC GTC ATA CAC TTG CTT CT-3′ and *GAPDH* (116 bp): F 5′-AGTGGCAAAGTGGAGATT-3′, R 5′-GTGGAGTCATACTCCAACA-3′.

### 2.2. Cell Culture and Transfection

TM4 cell, a mouse Sertoli cell line, was purchased from the ATCC biological resource center (Manassas, VA). The cells were cultured in Dulbecco's Modified Eagle's Medium (Gibco) containing 10% fetal bovine serum (*v*/*v*) and 100 *μ*g/mL of penicillin and 100 *μ*g/mL of streptomycin at 37°C with atmospheric conditions of 95% air and 5% CO_2_.

Cells plated in a 6-well plate were transfected with 3 *μ*g plasmids by Lipofectamine 3000 (Life Technologies) when 40%–60% confluence was reached. The cell culture medium was changed to a culture medium containing 2 *μ*g/mL puromycin 24 h later for 3 d. Subsequently, cells were kept in a culture medium containing 0.5 *μ*g/ml puromycin.

### 2.3. Co-IP and Western Blot Analysis

After the cells were harvested, cytoplasmic membrane and nuclear protein fractions were separated using the subcellular protein fractionation kit for cultured cells (Pierce, Life Technologies) as described [12]. Protein concentrations were determined by bicinchoninic acid protein assay. Subsequently, 500 *μ*L aliquots containing 500–1000 *μ*g of protein were subjected to immunoprecipitation using 5 *μ*g of rabbit polyclonal antibody, anti-AR (ab74272, Abcam, MA) and 5 *μ*g of rabbit polyclonal antibody, anti-AR (N-20, sc-816, Santa Cruz Biotechnology, CA) or 10 *μ*g of rabbit polyclonal antibody, and anti-caveolin-1 (N-20, sc-894, Santa Cruz Biotechnology, CA). After separation of immunoprecipitated proteins with the use of protein G Dynabeads (Life Technologies), pellets were resuspended in 30 *μ*L of sodium dodecyl sulfate loading buffer for Western blot analysis.

Proteins (20 *μ*g) were subjected to sodium dodecyl sulfate-polyacrylamide gel electrophoresis on 10% tricine-glycine gels and then transferred to polyvinyl difluoride membranes (Millipore). After blocking by incubation for 1 h at room temperature in 50 mM Tris-HCl (pH 8.0), 150 mM NaCl, and 0.1% Tween 20 (Tris-buffered saline (TBST)) containing 5% nonfat dry milk, membranes were incubated at 4°C with anti-AR antibody (rabbit polyclonal, 1 : 1000, Santa Cruz Biotechnology) overnight. Membranes were incubated for 1 h at room temperature with the second antibody in 5% nonfat dry milk in TBST after membranes were washed 3 times (5 min each) in TBST. Specific complexes were detected using the enhanced chemiluminescence system from GE Healthcare. After exposure, blots were stripped and assayed for histone deacetylase 1 (ab53091, Abcam, 1 : 1000) in the nucleus, pan-cadherin (ab22744, Abcam, 1 : 5000) for the membrane, and glycerol glyceraldehyde-3-phosphate dehydrogenase (GAPDH; V-18; Santa Cruz Biotechnology, 1 : 1000) for the cytoplasm as loading controls.

### 2.4. Immunofluorescence

TM4 cells plated on coverslips were preincubated in medium supplemented with stripped serum overnight. Cells were then washed and incubated in medium with 0.1% BSA before addition of testosterone at 10 nM. After 30 minute incubation, cells were fixed for 15 min with 4% formaldehyde, washed with PBS, treated 2 × 5 min with 30 mM glycine to quench fixation, and washed again with PBS. The coverslips were blocked using 5% bovine serum albumin (Sigma-Aldrich), incubated with primary anti-AR (ab74272, Abcam) antibody overnight, and rinsed and incubated with secondary antibody (1 : 500, Thermo Fisher) for 2 h at room temperature. The slides were then rinsed in PBS for 3 × 5 min and incubated for 5 min at room temperature with Hoechst 33342 (1 : 5000, Thermo Fisher). Cells were washed with PBS for 3 × 5 min again and then mounted with Prolong Gold Antifade Reagent (Thermo Fisher) for imaging.

### 2.5. Statistical Analysis

Data were presented as the mean ± SEM. The statistical significance of the differences between groups was determined by the independent *t-*test or a one/two-way ANOVA followed by a Fisher-protected least significant difference post hoc test. Statistical significance was considered when *P* < 0.05.

## 3. Results

### 3.1. Testosterone Induced Membrane Association of AR

**Figure 1:**
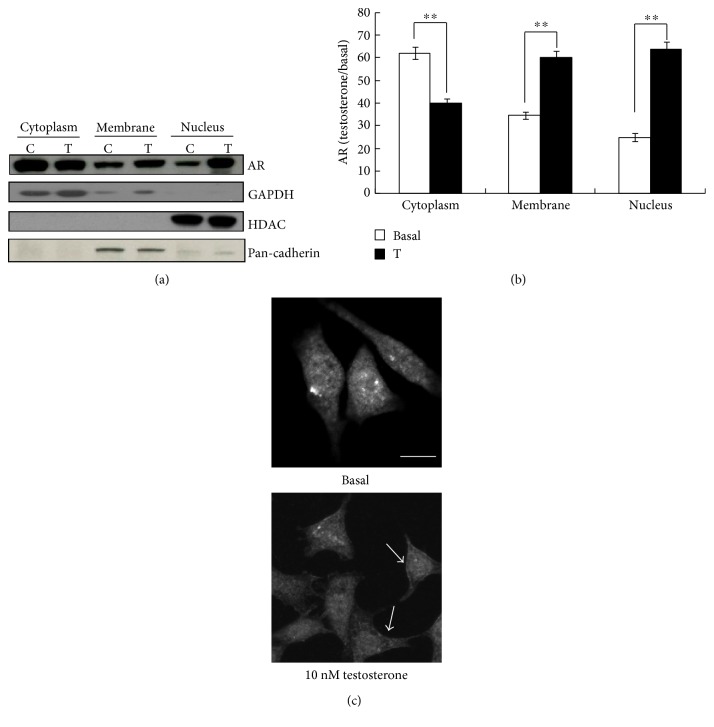
Testosterone induced membrane association of AR. (a) Representative Western blot analysis of AR levels in cytoplasmic, membrane, and nuclear proteins from cells incubated for 30 min with vehicle (control (C)) or 10 nM testosterone (T). (b) Semiquantitative analysis of AR levels in the subcellular fractions after exposure of the cells to vehicle (basal) or testosterone for 30 min. Data were expressed as the mean and SEM of the ODs of the AR band, multiplied by the testosterone-to-basal ratio of the respective mark proteins, GAPDH, HDAC, and pan-cadherin for cytoplasmic, nuclear, and membrane fractions. ^∗∗^*P* < 0.01 compared with that of the respective basal (*n* = 5). Confocal microscopy imaging of the effect of testosterone on AR trafficking. Cells were incubated with 10 nM testosterone for 30 min, before fixation and confocal microscopy examination. Bar = 50 *μ*m.

To verify the membrane association of the AR, we performed Western blot analysis on the subcellular protein fractions of TM4 cells expressing endogenous AR, shown as a 110 kDa band corresponding to the molecular weight of AR. As expected, 10 nM testosterone exposure 30 min significantly increased AR in the nucleus by 2.56 ± 0.02-fold (*P* < 0.01) (Figure 1(a)). A band corresponding to AR was also observed in the membrane protein fractions colocalized with pan-cadherin. One-way ANOVA also showed the significant effect of testosterone on AR levels in the membrane protein (*F* = 1280, *P* < 0.01) (Figure 1(b)).

To verify the translocation of AR under testosterone induction, which was exhibited by Western blot assay, we also performed immunofluorescence assay with the specific antibody against AR. As shown in Figure 1(c), under basal conditions, AR fluorescence was present predominantly in the cytoplasm. Consistent with the Western blot results, after 30 min incubation with 10 nM testosterone, fluorescence was observed in the nucleus, as well as the membrane (shown as the arrow).

### 3.2. Membrane AR Associated with Caveolin-1

**Figure 2:**
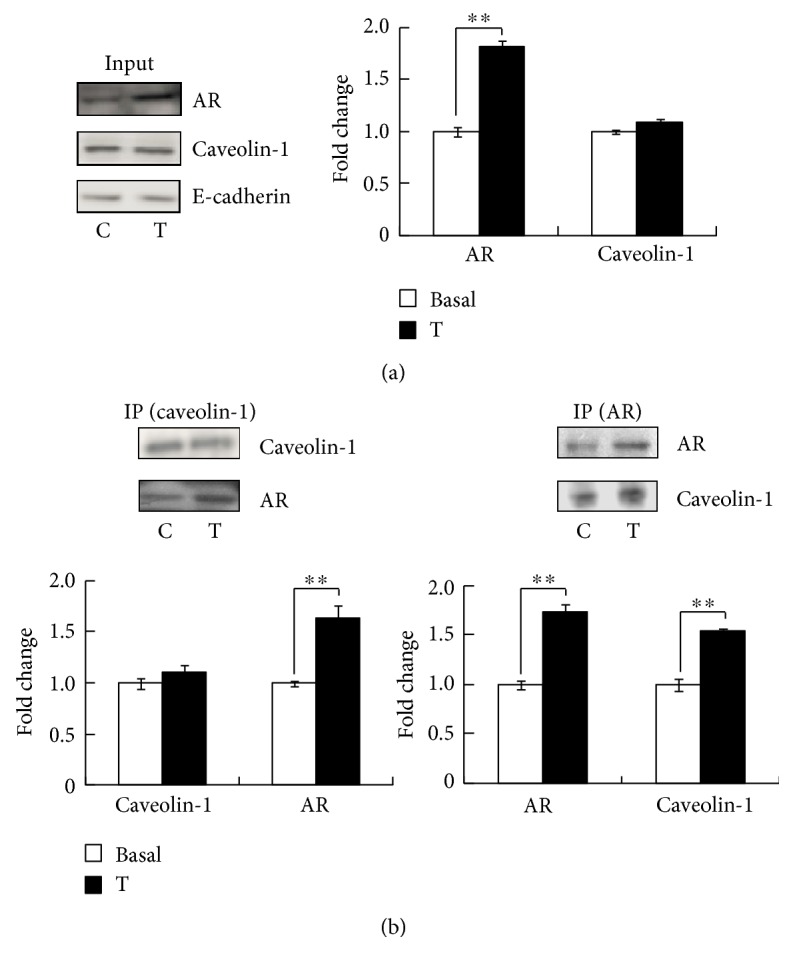
Membrane AR associated with caveolin-1. (a) AR and caveolin-1 translocation in membrane fraction under testosterone treatment in TM4 cells. (b) The film images showed representative Western blot analysis for caveolin-1 and AR in the membrane fraction proteins after immunoprecipitation by the anti-caveolin-1 antibody (left) or anti-AR antibody (right) in TM4 cells treated with 10 nM testosterone for 30 min. ^∗∗^*P* < 0.01.

To determine whether the membrane localized AR was associated with the caveolae protein caveolin-1, membrane fraction proteins from cells treated with vehicle (basal) or testosterone for 30 min were immunoprecipitated with the anti-caveolin-1 antibody or anti-AR antibody. Consistent with Figure 1, testosterone induced AR translocation to the membrane (Figure 2(a)). However, the expression of caveolin-1 in membrane fraction was unchanged (Figure 2(a)). As expected, in the protein fraction immunoprecipitated by the anti-caveolin-1 antibody, the density of the band of the membrane caveolin-1 remained unchanged because this protein was localized on the membrane. Testosterone increased the association of AR with caveolin-1 (Figure 2(b), left). Testosterone-induced membrane AR trafficking is shown in the fraction immunoprecipitated by the anti-AR antibody; in agreement with the data before, more AR in membrane fractions pull down more caveolin-1 (Figure 2(b), right). These data suggested that membrane AR was associated with caveolin-1.

### 3.3. Caveolin-1 Is Essential for AR Membrane Localization

**Figure 3:**
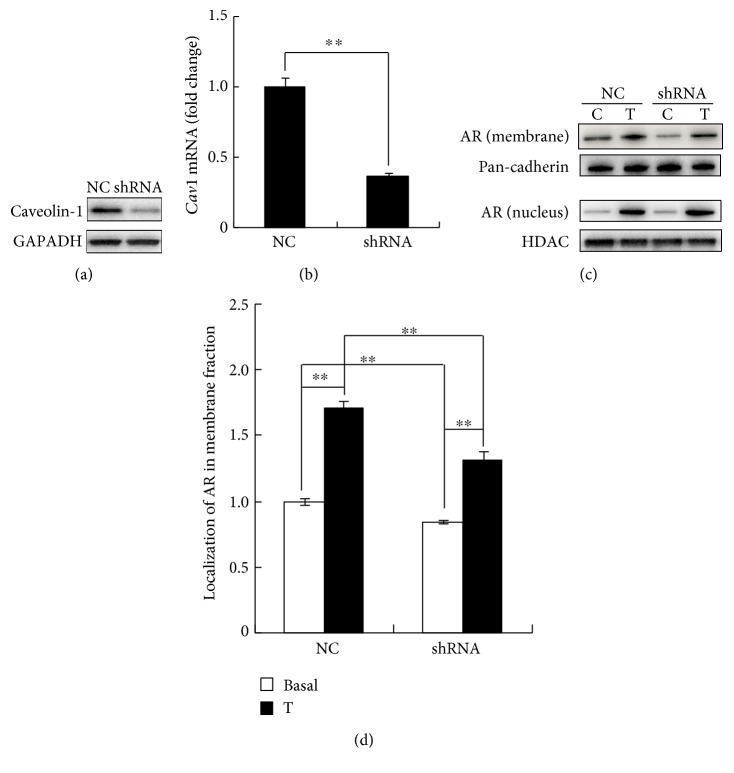
Knockdown of caveolin-1 decreased AR membrane localization and translocation. Results of representative Western blot analysis (a) and real-time PCR (b) show caveolin-1 knocked down by shRNA transfection. (c) Representative Western blot analysis indicating that AR trafficking under 10 nM testosterone treatment in membrane and nuclear fractions from cells transfected with negative control shRNA or caveolin-1 shRNA. (d) The graph shows the mean and SEM of AR expression in the membrane fraction from 4 experiments. Values are expressed as fold-change of the negative control basal. ^∗∗^*P* < 0.01.

To verify the immunoprecipitation data, caveolin-1 was knocked down by shRNA provided by Shanghai Genechem Co. Ltd. The results of Western blot analysis (Figure 3(a)) and real-time PCR (Figure 3(b)) indicated that protein caveolin-1 was decreased by 50% and that mRNA was knocked down by 60%.

In the cells transfected with negative control shRNA or caveolin-1 shRNA, knockdown of caveolin-1 does not affect AR nuclear translocation (Figure 3(c)); no significant difference could be drawn from the data analysis (data not shown). Consistent with the immunoprecipitation experiments, knockdown of caveolin-1 decreased AR localization to the membrane in basal condition (NC basal 1.00 ± 0.021 versus shRNA basal 0.84 ± 0.009, *P* = 0.004, *n* = 4) and prevented AR translocation after exposure to testosterone (NC testosterone 1.72 ± 0.053 versus shRNA testosterone 1.31 ± 0.075, *P* < 0.001, *n* = 4). And the ratios (testosterone/basal) in NC- and shRNA-treated cells were 1.72 ± 0.037 and 1.56 ± 0.043, respectively (*P* = 0.030).

### 3.4. 2-Bromopalmitate Prevented AR Translocation to Membrane

**Figure 4:**
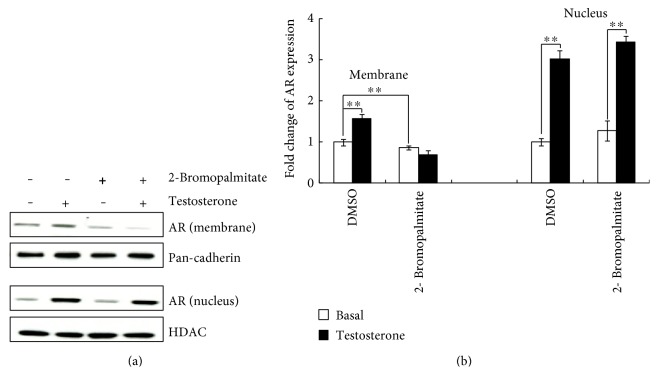
2-Bromopalmitate prevented AR translocation to membrane. (a) Representative Western blot images showing AR transport in the membrane and nuclear fraction proteins from cells preincubated with vehicle (DMSO) or 2-bromopalmitate for 2 h before exposure to 10 nM testosterone. (b) Pooled data from 3 individual experiments. Data are expressed as mean ± SEM, and the values are calculated from the fold change of DMSO basal. The concentration of 2-bromopalmitate used in this study is 50 *μ*M. ^∗∗^*P* < 0.01.

Palmitoylation of the cysteine residue in ER increases the physical association of ER with caveolin-1 [10]. We used the palmitoylation inhibitor 2-bromopalmitate (Sigma Aldrich) to inhibit palmitoylation of AR. Cells were preincubated with 50 *μ*M 2-bromopalmitate for 2 h before testosterone treatment. As shown in the Figure 4(a) and statistical analysis (Figure 4(b)), inhibition of palmitoylation exerted no effect on AR translocation to the nucleus. However, 2-bromopalmitate prevented AR membrane localization in basal condition (DMSO basal 1.00 ± 0.075 versus 2-bromopalmitate basal 0.86 ± 0.049, *P* < 0.01, *n* = 3) and completely blocked AR membrane trafficking (2-bromopalmitate basal 0.86 ± 0.049 versus testosterone 0.68 ± 0.126, *P* = 0.241).

## 4. Discussion

Androgens affect male fertility in various ways. The action of androgens on Sertoli cells regulates claudin-3 expression and the formation and maintenance of the blood-testis barrier (BTB) [13, 14], as well as meiosis, Sertoli-spermatid adhesion, and sperm release. The classical pathway is undoubtedly involved and dominant; nevertheless, the nonclassical pathway participates in these events. Recently, rapid signaling effects of testosterone have been increasingly recognized [15] and ascribed to membrane-localized AR [16]. However, the exact nature of testosterone-associated membrane proteins remains unclear, and the mechanisms required for membrane association are undetermined.

In the current study, using the subcellular fractionation kit and immunofluorescence, we found that physiological testosterone induced a rapid membrane association of AR within 30 min (Figure 1), and significant association could be detected within 5 min (data not shown). This rapid action is suggested to be a nonclassical signaling pathway. This finding also strongly proved membrane AR mediation of the nonclassical action of testosterone, which required no DNA amplification or protein synthesis.

Caveolin-1 is a protein that in humans is encoded by *Cav1* [17]. This scaffolding protein is the main component of caveolae plasma membranes found in most cell types. The caveolin family consists of 3 members, caveolin-1, caveolin-2, and caveolin-3. Caveolin-1, which reportedly interacts with AR [18], is a possible modulator of AR-mediated transactivation. A ChIP-qPCR study conducted by Hu et al. showed 2 binding sites of AR associated with caveolin-1 [19]. Our immunoprecipitation data indicated that membrane AR bonded to caveolin-1 and that testosterone promoted this association (Figure 2). We also evaluated the potential association of AR with caveolin-2 and caveolin-3; no positive association was indicated (data not shown). Knockdown of caveolin-1 decreased AR localization to the membrane and inhibited AR membrane translocation under testosterone treatment (Figure 3). These supported AR localization to the membrane by binding to caveolin-1.

The mechanism underlying the binding of AR to caveolin-1 needs further study. Nuclear receptors are ligand-activated factors sharing a common evolutionary history [20]. A signature, 9 amino acid motif (F(X6)LL sequence) was found to be highly conserved in the ligand-binding domains of all sex steroid receptors not including AR and identical in both human and mouse genes [10]. Although the sequence homology is scarce, AR contains the predicted s-palmitoylable cysteine residue as do other steroid hormone receptors [21]. This cysteine residue might be the main contributor to the membrane localization of these steroid hormone receptors [21].

AR sequence was further confirmed to be palmitoylable by analysis with CSS-Palm 4.0 (free available from: http://csspalm.biocuckoo.org/) [22]. AR contains putative sites that exhibit an increased chance for palmitoylation when analyzed with CSS-Palm 4.0 software; nevertheless, they were discarded because of protein conformation, disulfide bond formation, cysteine accessibility, and cysteine-metal coordination [21]. Our data showed that the palmitoylation inhibitor 2-bromopalmitate prevented AR localization to the membrane and completely blocked AR membrane trafficking after exposure to testosterone. As expected, this inhibitor exerted no effect on AR nuclear localization and trafficking, which indicated that AR localized to the nucleus is not palmitoylated. A lower concentration of 2-bromopalmitate (10 *μ*M) was also used to evaluate the effect of the inhibitor on AR translocation. We observed an inhibitory effect on testosterone-induced AR translocation, but the difference was not as significant as that of the 50 *μ*M inhibitor. The palmitoylation inhibitor potentially exerts an inhibitory effect on other palmitoylated proteins that are important for AR trafficking. In the study by Pedram et al. [10], AR was found palmitoylated in LnCaP prostate cancer cells, which express endogenous AR. A mutant of the putative palmitoylated cysteine in AR prevented AR membrane localization. To summarize, AR is associated with the membrane by binding with caveolin-1 through palmitoylation.

Palmitoylation appears to be a common feature for some steroid hormone receptors. Palmitoylation may be involved in numerous signaling protein plasma membrane localization [23, 24], yet this modification must be regarded as more than a simple mechanism. Owing to its reversible nature, palmitoylation is particularly important for modulating protein activation and deactivation [24, 25]. All of studies [10, 26] did not draw a conclusion that sex steroid promotes the receptor palmitoylation; instead of that, the steroids may induce depalmitoylation of the receptors. Once the membrane association is attained, depalmitoylation of many proteins immediately occurs. Depalmitoylation of AR allows protein-protein interaction, which is necessary for membrane AR signal transduction.

In the present study, we found that testosterone induced AR translocation to the membrane fraction. AR was localized to the membrane by association with caveolin-1 via palmitoylation, and testosterone promoted this association. Knockdown of caveolin-1 decreased AR membrane localization and inhibited AR translocation to the membrane after exposure to testosterone. This proved AR membrane localization and helped clarify the mechanism of AR membrane anchoring and enriched the nonclassical pathway of androgens. Further studies should be conducted to identify and investigate the signal molecules and transport proteins involved in AR translocation to the membrane fraction. These data could potentially be used to address the challenges of treating infertility due to defects in testosterone signaling.

## Figures and Tables

**Table 1:** The sequence of shRNA to caveolin-1.

ID	5′	Stem	Loop	Stem	3′
Cav1-RNAi-a	Ccgg	ccGCTTGTTGTCTACGATCTT	CTCGAG	AAGATCGTAGACAACAAGCGG	TTTTTg
Cav1-RNAi-b	aattcaaaaa	ccGCTTGTTGTCTACGATCTT	CTCGAG	AAGATCGTAGACAACAAGCGG	
